# Pulmonary Fat Embolism After Fat Grafting

**DOI:** 10.3390/diagnostics15243214

**Published:** 2025-12-16

**Authors:** Xin Lu, Huadong Zhu, Yi Li

**Affiliations:** Emergency Department, State Key Laboratory of Complex Severe and Rare Diseases, Peking Union Medical College Hospital, Chinese Academy of Medical Sciences and Peking Union Medical College, Beijing 100730, China

**Keywords:** fat embolism syndrome, echocardiography, fat grafting

## Abstract

Fat embolism syndrome (FES) is a clinical syndrome in which the obstruction of small blood vessels by fat emboli triggers a systemic inflammatory response, leading to organ dysfunction. Due to a lack of specific laboratory tests and physical examination, FES is clinically underdiagnosed. We report a case of a 39-year-old woman who presented with dyspnea that had developed after augmentation mammaplasty and vaginal tightening with autologous fat. Bedside transthoracic echocardiography (TTE) carried out in our emergency department evidently revealed right heart embolic material presumed to be fat. Based on echocardiography findings, combined with medical history and computed tomography pulmonary angiography images, a diagnosis of pulmonary fat embolism was made. This case presents valuable echocardiographic images and emphasizes the availability of bedside TTE in the diagnosis of fat embolism in a patient with dyspnea after plastic surgery, highlighting the value of bedside TTE in rapidly identifying pulmonary fat embolism.

**Figure 1:**
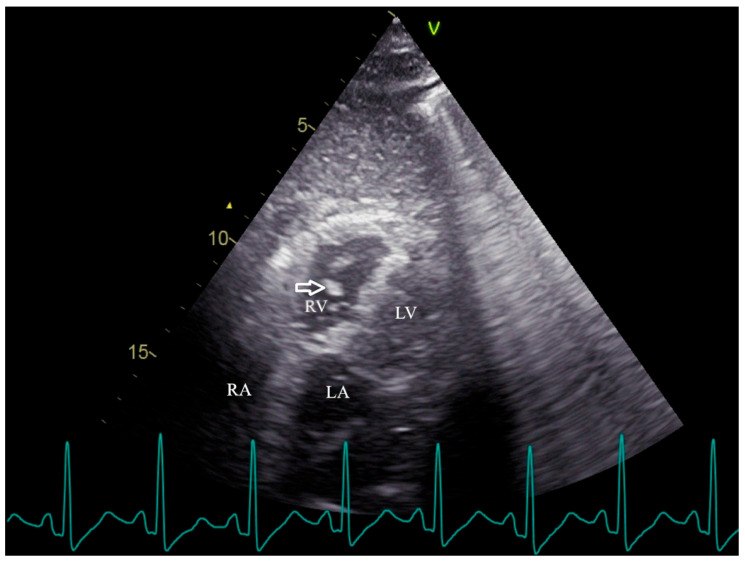
Transthoracic echocardiography reveals the mobile, irregular embolic material (arrow) presumed to be a fat mass within the right ventricle. RV, right ventricle; RA, right atrium; LV, left ventricle; LA, left atrium. Fat embolism (FE) was first recognized more than 150 years ago, describing the presence of fat globules within the circulatory system regardless of clinical symptoms [1]. Fat embolism syndrome (FES) is a clinical syndrome in which the obstruction of small blood vessels by fat emboli triggers a systemic inflammatory response, leading to organ dysfunction [2]. FES manifests 12 to 72 h after the initial insult and usually appears with multiorgan dysfunction, notably of the lungs, skin, and brain [2,3]. The classic triad involving hypoxemia, neurological dysfunction, and petechial rash is infrequent [4]. Dyspnea is the most common manifestation due to hypoxemia, affecting more than 75% of FES patients [2,3,5]. Due to a lack of specific laboratory tests and physical examination, FES is clinically underdiagnosed [4]. While transthoracic echocardiography (TTE) is commonly used to assess secondary signs of FES, direct visualization of mobile cardiac fat emboli remains rare. This case provides definitive TTE evidence of hyperechoic right ventricular masses, confirmed via computed tomography pulmonary angiography (CTPA) as fat-density emboli, highlighting a unique and underreported diagnostic scenario following elective cosmetic surgery. A 39-year-old woman presented to the emergency department with dyspnea. The patient underwent augmentation mammaplasty and vaginal tightening with autologous fat taken from the inner thighs one day ago; 15 min after the operation, she complained of sudden dyspnea with lip cyanosis. On physical examination, her oxygen saturation was 48% while breathing ambient air, blood pressure was 58/40 mmHg, heart rate was 130 beats per minute, and respiratory rate was 31 respirations per minute. Immediate interventions were initiated, including supplemental oxygen via nasal cannula, aggressive intravenous fluid administration, and a dopamine infusion for hemodynamic support. Following these measures, the patient’s blood pressure improved to 95/60 mmHg. Subsequent arterial blood gas (ABG) analysis yielded the following results: pH 7.23, PaCO_2_ 37 mmHg, PaO_2_ 86 mmHg, and HCO_3_^−^ 15.1 mmol/L, with a lactate level of 2.8 mmol/L. The patient was subsequently transferred to the resuscitation room in the emergency department for continued critical care management. Cardiopulmonary examination was unremarkable on admission. Laboratory findings were notable for a serum D-Dimer level of 15.57 mg per liter (reference range, 0 to 0.55), a troponin I level of 1.586 μg per liter (reference range, 0 to 0.056), a white cell count of 19,110 per cubic millimeter (reference range, 3500 to 9500), a hemoglobin level of 146 g/L, and a platelet count of 78,000 per cubic millimeter. Arterial blood gas analysis revealed mild hypoxemia (PaO_2_ 74.1 mmHg in room air) and a lactate level of 2.1 mmol/L. TTE revealed right atrial and ventricular enlargement. Additionally, multiple complex, mobile, irregular lesions were observed within the right ventricle. Based on the patient’s clinical presentation, these lesions were presumed to represent fat masses (arrows in Figure 1 and Video S1), rather than thromboemboli. Fat emboli typically appear as strongly hyperechoic foci with heterogeneous internal echotexture and demonstrate high mobility within the cardiac chambers [6,7]. In contrast, thromboemboli are generally iso- to hypoechoic, exhibit low mobility with frequent attachment to the endocardial wall, and present a more homogeneous morphology [8,9,10]. The right ventricle was mildly dilated (anteroposterior diameter 33 mm, transverse diameter 42 mm) with normal systolic function, evidenced by a tricuspid annular plane systolic excursion (TAPSE) of 20 mm and a right ventricular fractional area change (RVFAC) of 50%. The estimated pulmonary artery systolic pressure was 30 mmHg, derived from a tricuspid regurgitation velocity of 2.5 m/s. The inferior vena cava measured 12 mm in diameter with >50% respiratory collapse. The left ventricle was of normal size with preserved systolic function. CTPA demonstrated bilateral pulmonary artery filling defects (arrows, Figure 2). The differentiation of these lesions as fat embolism was critically informed by their CT attenuation values. The lipid composition of fat emboli confers a characteristically low CT attenuation, typically ranging from –50 to –150 Hounsfield units (HU). In contrast, thromboemboli, composed of clotted blood, exhibit significantly higher attenuation, with acute and chronic pulmonary thromboemboli averaging approximately 33 HU (95% confidence intervals: 26–41) and 87 HU (95% confidence intervals: 66–107), respectively [11,12]. In the present case, the measured mean attenuation of the lesions was approximately –55 HU. This value is definitely within the diagnostic range for fat and is congruent with the attenuation of subcutaneous fat (–95 HU) in the same imaging plane, providing definitive imaging evidence for pulmonary fat embolism over thrombotic disease. Furthermore, no thrombosis was observed in deep venous ultrasound of both lower extremities. Integrating the acute post-procedural presentation with imaging findings, the definitive diagnosis is pulmonary fat embolism. This is distinguished from other acute syndromes by its pathognomonic features: the hyperacute temporal link to fat grafting, the direct visualization of hyperechoic, mobile right heart masses on TTE, and the confirmation of fat-density (–55 HU) filling defects on CTPA. Critical alternative diagnoses are systematically excluded: pulmonary thromboembolism by the absence of deep venous thrombosis and the embolic material’s fat attenuation; amniotic fluid embolism by the non-peripartum context; infective endocarditis by the lack of systemic infection and the non-valvular, mobile nature of the masses; and cardiac tumors by their typical solitary, soft-tissue appearance. Other systemic causes fail to explain the intravascular emboli or the profound hypoxemia preceding collapse. Thus, pulmonary fat embolism remains the only entity that cohesively explains the entire clinical and radiological picture. The patient received a single 40 mg dose of methylprednisolone and was initiated on anticoagulation with enoxaparin. Two days later, anticoagulation was discontinued due to enlargement of the ecchymosis near the surgical sites and a drop in hemoglobin (from 146 g/L to 72 g/L). She recuperated gradually under supportive care. A follow-up echocardiography 1 month after discharge showed no sign of residual fat or other symptoms. FES is a rare life-threatening clinical syndrome, categorized into three clinical types, including pulmonary, cardiac, and neurologic types [3]. Gurd first described the diagnostic criteria for FES in 1970 [13], which were refined by Wilson in 1974 [14], including major clinical features such as respiratory distress, neurological symptoms, and petechial rashes. Subsequently, Lindeque et al. incorporated hypoxemia into the diagnostic criteria [15]. Schonfeld et al. established a diagnostic system with 5-point cutoff values [16]. The aforementioned criteria were established nearly 40 years ago, lacking imaging evidence from echocardiography or CTPA [13,14,15,16]. At present, the diagnostic methods of FES vary, including clinical manifestation, imaging, and histopathology. However, a gold standard diagnostic test remains lacking [12,17,18]. In our case, fat emboli were visualized within the right heart, confirming the diagnosis of FES. This demonstrates the value of bedside TTE in critically ill patients with suspected FE. FES typically results from fractures of the pelvis or long bones, and as many as 30% of these patients will develop FES [2]. On a smaller scale, histological FE is associated with pancreatitis, diabetes, cardiopulmonary bypass, osteomyelitis, severe burns, sickle cell anemia, lipid parenteral infusion, and liposuction [3,5]. As plastic surgery becomes increasingly popular, such embolic events are a rare but serious complication of liposuction and fat grafting [19]. It is usually asymptomatic, but in 1% to 5% of patients, macroscopic fat emboli composed of abundant adipocytes obstruct the pulmonary circulation, precipitating clinical outcomes resembling pulmonary thromboembolism [1,18]. In our case, with the patient presenting with respiratory distress and hypotension, pulmonary embolism (PE) was suspected. However, the clinical scenario alone is insufficient to distinguish pulmonary thrombotic embolism (PTE) from FES. CTPA has become the first-line imaging test for suspected PE [20,21], and some studies have examined common and unusual CT findings to identify FES and PTE [12]. A pulmonary imaging study involving 181 patients with FES found that 31 patients showed filling defects with negative mean attenuation values on CTPA [18]. However, the CT imaging features of fat embolism are non-specific and demonstrate significant overlap with those of PTE [12]. A study revealed that intensity volume and frequency shift with Doppler ultrasonography contribute to discriminating thrombi, fat, or marrow emboli [8]. The echocardiography-detectable right heart thrombus usually manifests as particulate matter, discrete, high acoustic densities that move within the cardiovascular system [6]. Particulate matter, such as fat, air, or marrow emboli, differs in appearance from the swirling, smoke-like spontaneous echo contrast, which represents low-flow red blood cell aggregates associated with hypercoagulable states [6,9]. Although these echocardiographic parameters may be helpful in identifying the source of the embolus, they have not been widely applied to clinical practice. A systematic review of reported cases showed that 24/95 (25.3%) of patients with FE following fat grafting underwent TTE [19]. Among these, the direct visualization of mobile hyperechoic fat globules within the right ventricle was distinctly uncommon. Echocardiographic findings were predominantly indirect signs such as pulmonary hypertension, reduced ejection fraction, patent foramen ovale, or non-specific hyperechoic floating dots [7,9,19]. Furthermore, our echocardiogram allowed the visualization of fat emboli in the right ventricle. Therefore, bedside TTE should be promptly performed to facilitate the identification of the correct etiology in a patient with dyspnea after plastic surgery. In contrast to CTPA, echocardiography may be more favorable, as it is noninvasive and does not employ radiation and contrast material, irrespective of age, pregnancy, or renal function [22]. With the implementation of echocardiography for the diagnosis of suspected FES, it will probably facilitate early recognition and prompt management in patients with respiratory distress symptoms induced by macroscopic fat embolism. In conclusion, echocardiography is an invaluable and convenient bedside tool among patients with sudden onset respiratory symptoms following surgery or trauma and is suggested to be promptly performed to correctly distinguish pulmonary fat embolism from thrombotic embolism. Definitive strategies for the treatment of FES remain elusive [5]. Patients with suspected FES often warrant admission to a critical care environment; therefore, our patient was immediately transferred to the resuscitation room [23]. Hypoxemia, often first identified through pulse oximetry monitoring in pulmonary fat embolism, should be managed initially with supplemental oxygen therapy [5,24]. To maintain adequate oxygenation, critically ill cases may even require mechanical ventilation, along with fluid infusion and vasoactive agents for circulatory support [23]. Over the decades, multiple pharmacological interventions for FES have been investigated, such as corticosteroids and heparin [5,23,25]. Previous studies of FES have shown improvements in the respiratory function and hemodynamic status with steroid administration [24,25]. However, due to the potential adverse effects of corticosteroids, such as secondary infections and delayed wound healing, the routine use of steroids to prevent FES is not generally recommended [5,25]. In light of these risks, corticosteroids were administered only once in our case. Heparin has been suggested as a treatment for FES by enhancing lipase activity to increase the clearance of intravascular lipids [3]. Moreover, the use of heparin, which can increase bleeding, is very limited in treating trauma or postoperative patients [5]. As demonstrated in our case, the patient developed an expanding surgical site hematoma with a marked drop in hemoglobin levels after two days of enoxaparin administration, leading to the discontinuation of anticoagulation therapy. A systematic review demonstrated that the overall mortality rate among patients with FES following fat grafting was markedly higher than that associated with traumatic FES (34.3% versus 7–10%) [19,26]. A review on pulmonary fat embolism following fat grafting showed that about 69% of patients experience symptoms within 24 h after surgery and that lesions on chest CT resolve 7 to 10 days after hospitalization [23]. Given the absence of specific treatments and the high mortality associated with pulmonary fat embolism, prompt diagnosis and the initiation of supportive care are critical [19].

**Figure 2:**
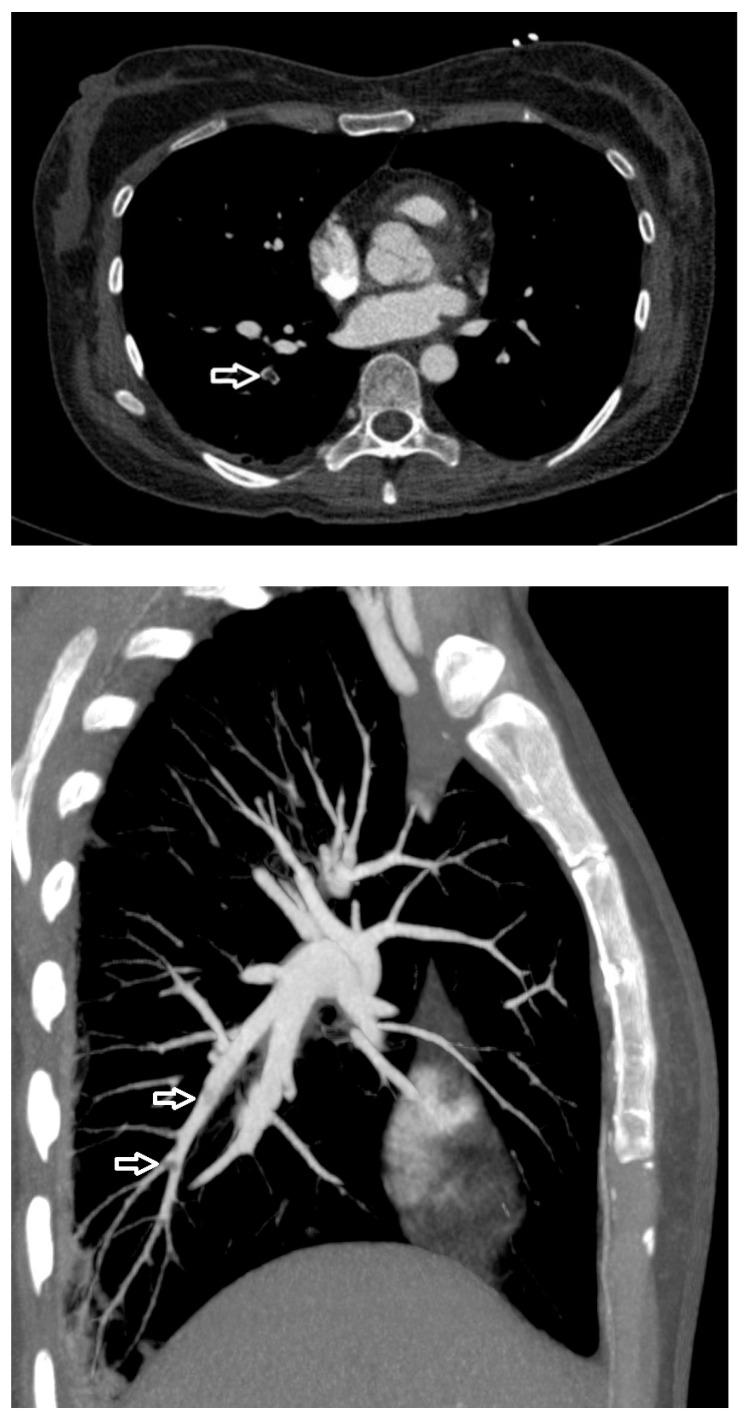
Computed tomography pulmonary angiography image shows filling defects (arrows) in the pulmonary artery, with a CT value of about −55 Hounsfield units (HU).

**Figure 3:**
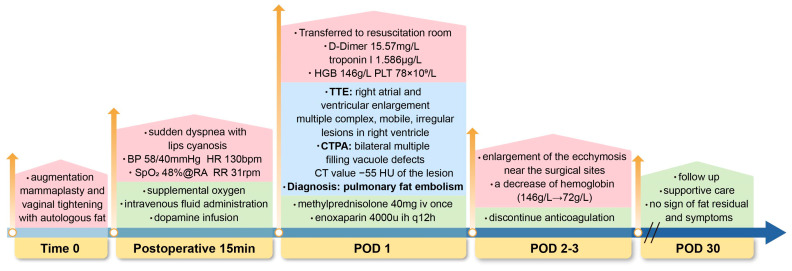
Timeline of diagnosis and management in a case of pulmonary fat embolism after fat grafting. Time 0 (End of surgery); POD, postoperative; BP, blood pressure; HR, heart rate; bpm, beats per minute; SpO_2_, oxygen saturation; RA, room air; RR, respiratory rate; rpm, respirations per minute; HGB, hemoglobin; PLT, platelet; TTE, transthoracic echocardiography; CTPA, computed tomography pulmonary angiography; HU, Hounsfield units; mg, milligram; iv, intravenous; ih, subcutaneous; u, unit; q12h, every 12 h; g/L, gram per liter.

## Data Availability

All data concerning the case are presented in this manuscript.

## Supplementary Materials

The following supporting information can be downloaded at https://www.mdpi.com/article/10.3390/diagnostics15243214/s1. Video S1: Transthoracic echocardiography shows the fat emboli within the right ventricle.

## Abbreviations

The following abbreviations are used in this manuscript:

ABG	Arterial blood gas
CTPA	Computed tomography pulmonary angiography
FE	Fat embolism
FES	Fat embolism syndrome
HU	Hounsfield units
LA	Left atrium
LV	Left ventricle
PE	Pulmonary embolism
PTE	Pulmonary thrombotic embolism
RA	Right atrium
RV	Right ventricle
RVFAC	Right ventricular fractional area change
TAPSE	Tricuspid annular plane systolic excursion
TTE	Transthoracic echocardiography
